# Anteroposterior Combined Surgery of a Rare Massive Epithelioid Hemangioendothelioma at the Cervicothoracic Junction

**DOI:** 10.7759/cureus.43032

**Published:** 2023-08-06

**Authors:** Hidayet Safak Cine, Ece Uysal, Salim Senturk, Gülnihal Ay, Basak Caner

**Affiliations:** 1 Neurosurgery, Istanbul Medeniyet University, Prof. Dr. Suleyman Yalcin City Hospital, Istanbul, TUR; 2 Neurological Surgery, Prof. Dr. Cemil Tascioglu City Hospital, Istanbul, TUR; 3 Neurosurgery, Memorial Spinal Center, Istanbul, TUR; 4 Pathology, Istanbul Medeniyet University, Göztepe Research and Training Hospital, Istanbul, TUR

**Keywords:** spinal tumor, pediatric neurosurgery, epitheloid hemangioendothelioma, craniocervical junction, combined surgery

## Abstract

Epithelioid hemangioendothelioma is a rare mesenchymal tumor of vascular endothelial origin. Non-soft tissue epithelioid hemangioendothelioma can also be seen in different organs. Although chemotherapy has been used in some patients, complete surgical removal of the tumor tissue has proven to be the most durable solution.

A 15-year-old female patient was admitted to our institution with right arm and neck pain. The patient complained of numbness and weakness in the right hand. Computerized tomography indicated an expansile lesion exhibiting osteolytic features located predominantly on the right side of the corpus, pedicle, lamina, and lateral processes of the C7-T1 vertebra. The patient underwent a surgical procedure involving the application of a bilateral C4-5-6 lateral mass screw, left C7-T1 pedicle screw, and bilateral T2-3 pedicle screw and fusion. The complete residual neoplasm was surgically removed during the procedure.

Due to the rarity of epithelioid hemangioendothelioma, the existing literature on this topic is confined to case reports, supplemented by a small number of retrospective descriptive case series that aimed to improve our understanding of the clinical, pathological, and molecular features of the condition, as well as to guide potential treatment strategies.

## Introduction

Epithelioid hemangioendothelioma, a rare borderline tumor consisting of epithelioid, endothelial, and dendritic cells, was first reported by Weiss-Enzinger in 1982 [1]. Its estimated prevalence is 1:1,000,000 in the general population [2]. Non-soft tissue epithelioid hemangioendothelioma can also be seen in different organs. Although the liver is the most commonly affected organ, the tumor can develop anywhere with visceral or soft tissue [3].

Epithelioid hemangioendothelioma is a rare mesenchymal tumor of vascular endothelial origin. In general, it may cause nodular or diffuse involvement in the liver. The nodular type is usually seen in the earlier stages of the disease, and in advanced stages, the nodules have been found to merge into diffuse involvement [4]. However, it can occur in the spinal canal, bladder, and lungs, where there is vascular endothelium. Compared to other malignancies, it has a less aggressive course than angiosarcoma. Metastasis is detected in 27% of cases and can be seen in the liver, lungs, regional lymph nodes, spleen, urinary system, and bones [5]. The clinical manifestations of the disease are non-specific. The diagnosis is mainly conducted radiologically and pathologically. In biochemical tests, serum alkaline phosphatase was elevated in 70% of patients, while carcinoembryonic antigen levels were within normal limits [4].

Histologically, the tumor consists of dendritic or epithelioid cells within the fibrous stroma. These cells typically spread within small veins, forming groups. Positive dendritic and/or epithelioid cells regarding endothelial cell markers confirm the diagnosis [5].

There is no established therapeutic algorithm. Although chemotherapy has been administered in some patients, it has been reported that the most durable solution is complete surgical removal of the tumor tissue [3-5]. This article reports the case of a 15-year-old girl with massive hemangioendothelioma at the cervicothoracic junction.

## Case presentation

Clinical summary

A 15-year-old female patient was admitted to our institution with right arm and neck pain. The nature of the pain was persistent without any change even at rest. The patient complained of numbness and weakness, particularly under the right arm’s elbow and in the third, fourth, and fifth fingers. Manual Muscle Testing was observed in grade 2, indicating an impairment in muscle strength for the flexion of the phalanx in the right hand. Deep tendon reflexes in the upper and lower extremities were not increased. Furthermore, there were no pathological reflexes.

**Figure 1:**
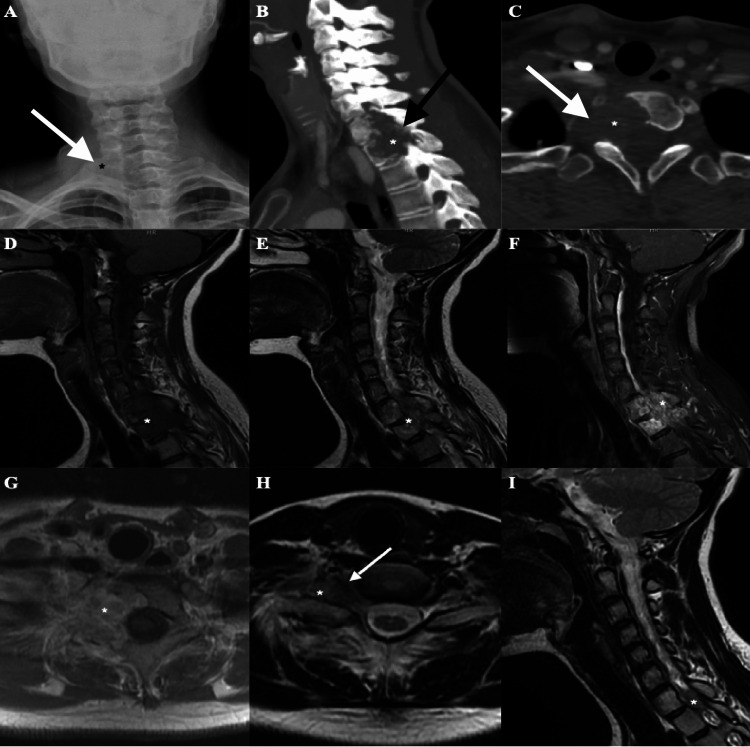
Preoperative images of the patient. (A) The cervical anteroposterior radiograph revealed the presence of an osteolytic lesion that appeared to be casting a shadow on the soft tissue on the right side. (B) The sagittal computed tomography (CT) and (C) axial CT scan revealed that the lesion had infiltrated the C7-T1 region and resulted in osseous destruction. The magnetic resonance imaging (MRI) findings indicated that the lesion exhibited (D) hypointensity on T1-weighted images, (E) hyperintensity on T2-weighted images, and (F) demonstrated significant enhancement on contrast images. The axial MRI images revealed that the neoplasm infiltrated the (G) soft tissue and the (H) right vertebral artery. (I) The patient’s MRI image reveals a tumor with an intradural onset two years ago. *: the tumor. White arrow: right vertebral artery.

Bladder and rectal functions were normal. The patient’s medical history revealed no previous illness, alcohol consumption, or smoking. The patient’s family history did not include any identifiable neurological disease. A series of radiological images of the cervical spine were obtained. A lesion with osteolytic features was identified on the cervical radiograph of the patient (Figure 1A). Computed tomography (CT) scan denoted the presence of an expansile lesion with osteolytic features located predominantly on the right side of the corpus, pedicle, lamina, and lateral processes of the C7-T1 vertebra (Figures 1B, 1C). A heterogeneous, expansile, and sclerotic lesion without calcification was observed as hypointense on T1-weighted and hyperintense on T2-weighted magnetic resonance imaging (MRI) (Figures 1D, 1E). In addition, the lesion displayed intense contrast enhancement (Figure 1F). The lesion had spread toward the pleural apex on the right side (Figure 1G) and had infiltrated the posterior region of the V1 segment of the right vertebral artery (Figure 1H). Despite the absence of apparent spinal cord compression, the demarcation between the spinal cord and the neoplasm was effaced. Upon reviewing the patient’s medical records, the MRI images showed a smaller tumor that had gone unnoticed by radiologists approximately two years ago (Figure 1I).

The positron emission tomography-computed tomography (PET-CT) findings indicated the primary tumor and did not reveal any additional metastatic lesions. Digital subtraction angiography revealed left vertebral artery dominance, but embolization could not be performed because no vascular structure feeding the tumor was found. The neoplasm was categorized as Ennecking G2T2M0 (2B) and Weinstein-Boriani-Biagini (WBB): 7-11 ABCD-F [6,7].

Hematological and biochemical blood parameters, urine vanillylmandelic acid analysis, and hemoglobin electrophoresis were within normal ranges. The electromyography assessment revealed noteworthy engagement of the C8 and T1 roots on the right side.

**Figure 2:**
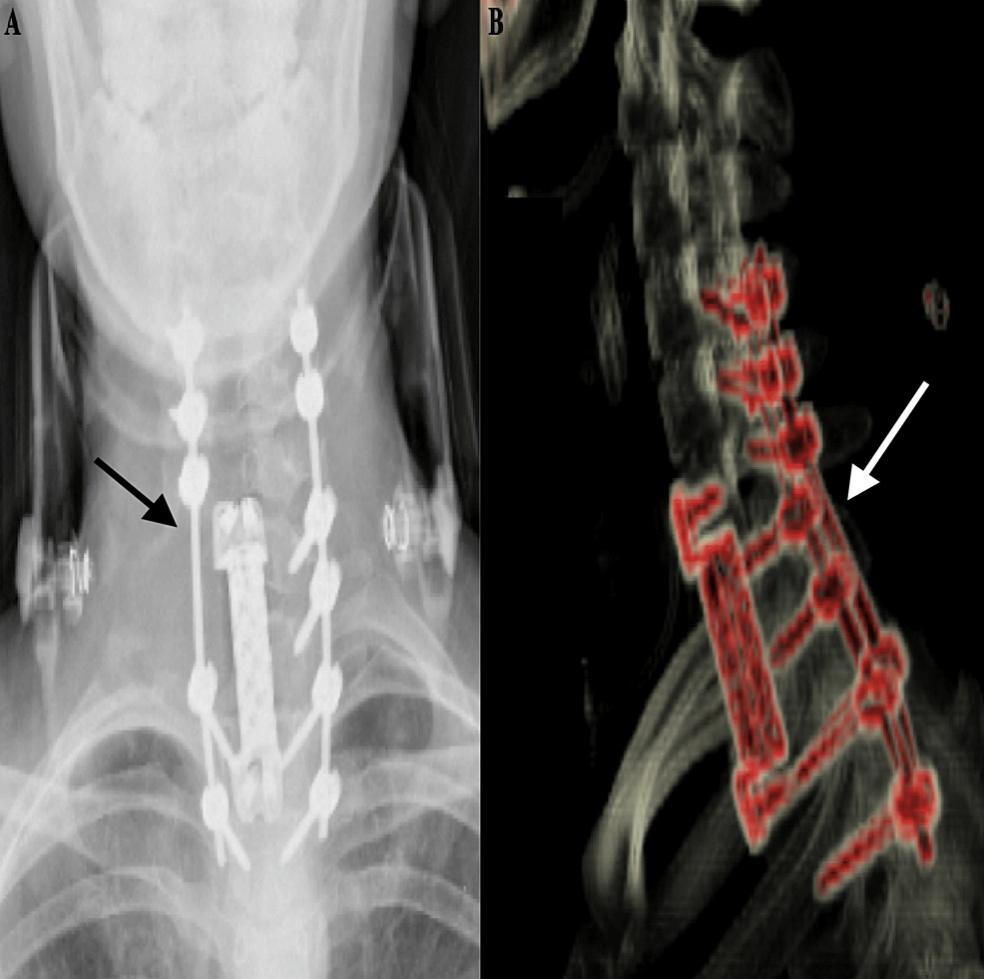
(A) Postoperative anteroposterior radiograph. (B) Postoperative three-dimensional computed tomography image.

A combined surgical procedure was planned for the patient. Before the operation, the thoracic surgeon conducted a video-assisted thoracoscopic surgery to examine the lung apex for any signs of tumor invasion. No such invasion was detected. The patient underwent a surgical procedure involving the application of a bilateral C4-5-6 lateral mass screw, left C7-T1 pedicle screw, and bilateral T2-3 pedicle screw and fusion (Figure 2).

During the insertion of the rod, the left C6 screw was extracted. The neoplasm was removed to the maximum extent feasible. The residual tumor tissue in the anterior region was retained for the subsequent surgical intervention. The tumor exhibited adhesion to the cervical roots, caused erosion of the bone with varied consistency, and was accompanied by significant hemorrhage (500 mL), as observed at the macroscopic level. The second surgical procedure involved the implementation of a C7-T1 corpectomy utilizing a cervicothoracic incision and a C6-T2 self-plated distractable cage. The complete residual neoplasm was surgically removed during the procedure.

**Figure 3:**
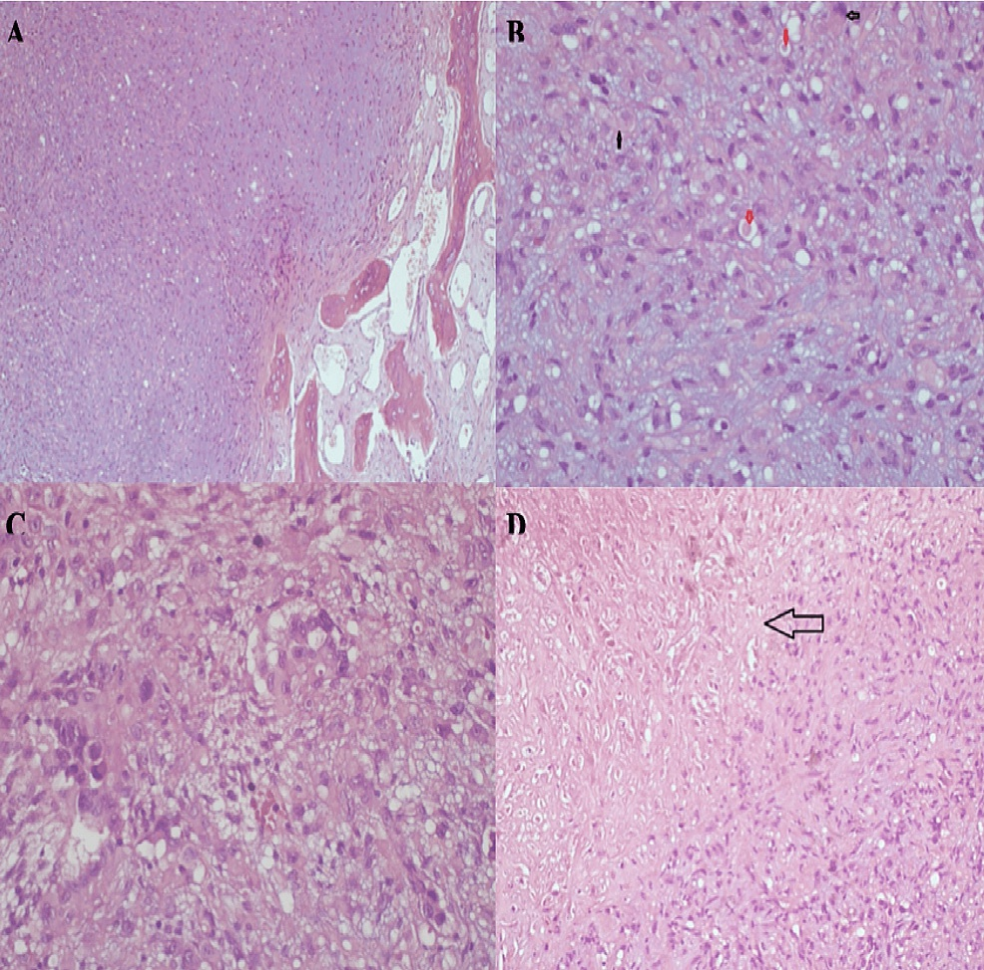
Histopathological findings of epitheloid hemangioendothelioma. (A) Hematoxylin-eosin staining (H&E) at 10× magnification: neoplastic proliferation with myxo hyalinized stroma alongside degenerated bone trabeculae. (B) H&E at 40× magnification: black arrow: epithelioid-looking atypical cells with large eosinophilic cytoplasm, red arrow: intracytoplasmic clear vacuoles, some of which contain erythrocytes. (C) Epithelioid appearance with extensive eosinophilic cytoplasm. (D) Area of necrosis.

**Figure 4:**
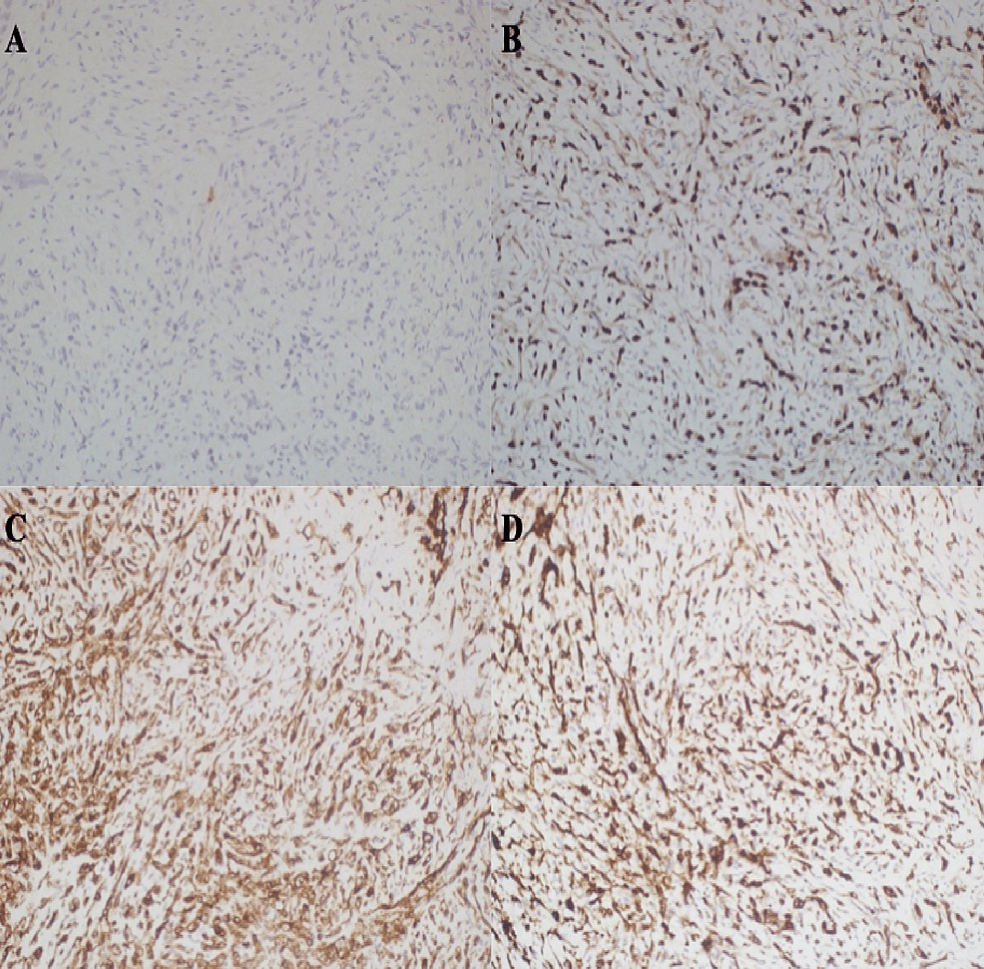
(A) Pan-CK (-), (B) ERG (+), (C) CD31 (+), and (D) CD34 (+).

The pathology findings indicated the presence of pleomorphism, characterized by areas of hemorrhage, bone trabeculae containing normal bone marrow, cord-like structures with myxo hyalinized stroma, occasional large eosinophilic cytoplasm, moderate pleophysis, and atypic cartilage and adipose-connective tissue. Clear vacuoles containing erythrocytes were observed within the intracytoplasmic region (Figure 3). The immunohistochemical findings indicate that CD34 and CD31 were strongly positive in a diffuse pattern, while D2 40 exhibited weak positivity (Figure 4). ERG was positive, whereas pan-CK, EMA, S100, and SATB2 were negative. The pathology report indicated that the tumor was identified as epithelioid hemangioendothelioma.

**Figure 5:**
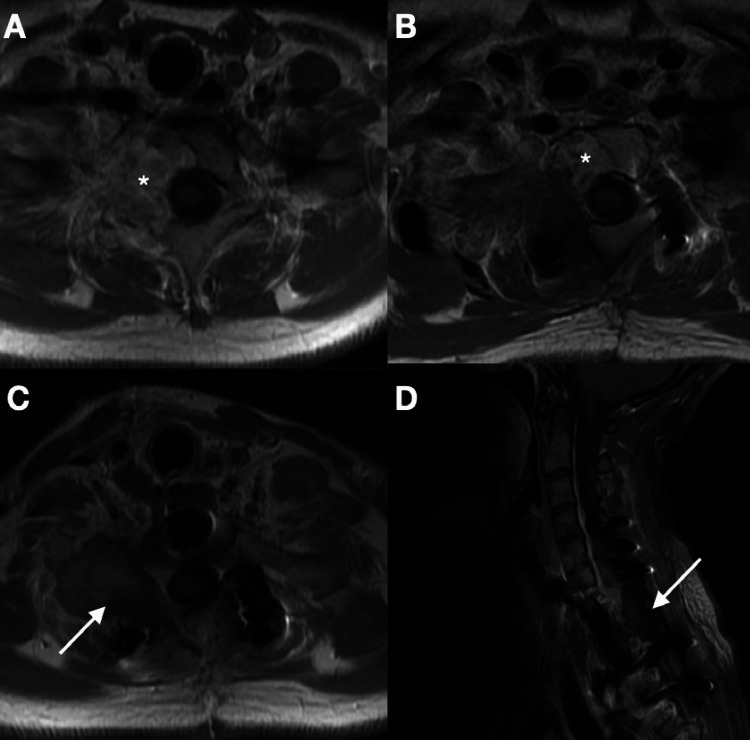
Postoperative images of the cervicothoracic junction tumor. (A) Axial preoperative magnetic resonance imaging (MRI). (B) After the first operation with the posterior approach, the axial MRI image showed wide resection of the tumor in the posterior area. Axial MRI (C) and sagittal MRI (D) showing complete excision of the tumor after anterior surgery, the second surgery. *: the tumor. White arrow: tumor lodge.

The initial MRI scans following the patient’s surgery showed complete and thorough removal of the tumor (Figure 5). Following the surgical intervention, almost 70% improvement was observed in the patient’s neurological condition. The patient declined any additional therapy following the surgical intervention. During the average one-year follow-up period, neither local tumor reappearance nor instrument failure was detected. No evidence of systemic metastasis was observed.

## Discussion

Epithelioid hemangioendothelioma is a rare vascular neoplasm originating from various anatomical locations, exhibiting significant diversity in clinical manifestation and prognostic results. The clinical severity of the condition is moderate, falling between the benign characteristics of hemangiomas and the markedly aggressive behavior of angiosarcoma [8]. Hemangioendothelioma is a type of tumor characterized by its angiocentric nature and has the potential to metastasize. It can manifest at any point in an individual’s lifespan. This phenomenon is most prevalent in elongated bones. In contrast, the manifestation of lesions in the spinal region is comparatively infrequent [9].

Because of the tumor’s rarity, the existing literature on this topic is confined to case reports, supplemented by a small number of retrospective descriptive case series that aims to enhance our understanding of the clinical, pathological, and molecular features of the condition, as well as to guide potential treatment strategies [10].

The method of removing a malignant tumor in the spine is to first try to remove the tumor as much as possible if the tumor can be excised. As metastasis is possible, screening for metastatic lesions, especially in tissues such as the liver and lungs, is necessary. It has also been seen that chemotherapy is given to these metastatic tumors [11]. Due to their localization, spinal bone tumors are not very susceptible to marginal excision except for wide excision and total removal. Therefore, after the widest possible tumor removal is achieved, radiotherapy can be used to reduce local recurrence [12].

In this case report, we presented a cervical spinal epithelioid hemangioendothelioma. The tumor was presumed to be malignant in the radiological images due to the invasion of the surrounding tissues, rapid progression, and intense contrast involvement. According to the Ennecking classification, a malignant tumor with high grade (G2), soft tissue, and extra-compartmental local invasion (T2), but no lesions were detected on PET-CT and internal organ metastasis scan (M0) was classified as 2B. Regarding Ennecking classification, effective adjuvant treatment was recommended for 2B classification in addition to wide en bloc resection. Therefore, it was necessary to remove the entire tumor extensively. According to the Weinstein, Boriani, Biagini (WBB) anatomical classification, the bone localization of the tumor was 7-11 due to the involvement of the right-located corpus, pedicle, lamina, transverse processing, and superior articular process. It was also classified as ABCD-F because of extraosseous soft tissue, intraosseous superficial and deep structures, extradural compartment, and vertebral artery invasion. Due to the anatomical localization of the tumor, vertebrectomy, right sagittal resection, and posterior arch resection with the oar of the WBB classification were considered necessary. Therefore, anterior and posterior interventions were planned to reach the entire tumor. As the patient had a neurological deficit at admission, a posterior intervention was performed to detect cervical nerve invasion and decompress the tumor by removing it. Posterior instrumentation was planned because of the cervicothoracic junction localization to prevent instability that may occur in addition to tumor excision. Therefore, fixation and fusion between C4 and T3 were performed so that the tumor between C7 and T1 went up three levels to the upper and two to the lower distance. The corpectomy procedure was done to address the residual tumor during the anterior surgery. The placement of the anterior cage and fixation serves a dual purpose of safeguarding the posterior instruments and averting kyphotic deformity. Given the necessity of prioritizing posterior surgery, the optimal choice for anterior support was subsequently selected. Autograft was not considered the primary option due to the potential for autograft shrinkage resulting from the administration of high-dose radiotherapy following the surgical procedure. The utilization of a cage for the patient was deemed necessary due to the potential risk of inducing deformity and subsequent failure of the posterior instrument.

While biopsy and pathology outcomes hold significance in primary vertebral tumors, particularly in the pediatric population, prioritizing decompression is crucial in instances of significant neurological function loss during the patient’s initial clinic admission. Otherwise, it should be acknowledged that the impairment of neurological function may be irreversible and result in unfavorable outcomes. For this particular type of neoplasm, it is recommended that the excision procedure be performed with a wide margin, extending as far as feasible. Following vertebral column resections, it is imperative to strategize for combined surgery either in a single session or across multiple sessions to mitigate the risk of instability and instrument failure.

## Conclusions

Due to the rarity of cervicothoracic epithelioid hemangioendothelioma, the existing literature on this topic is confined to case reports, supplemented by a small number of retrospective descriptive case series that aims to enhance our understanding of the clinical, pathological, and molecular features of the condition, as well as to provide guidance on potential treatment strategies. The clinical severity of the condition is moderate, falling between the benign characteristics of hemangiomas and the markedly aggressive behavior of angiosarcoma, and therefore should be treated cautiously. Surgery planning for primary spinal tumors should be based on the tumor’s pathology and the localization of the tumor in cases where wide resection is necessary. To mitigate postoperative instability, it is imperative to undertake essential instrumentation planning.
